# Differential Diagnosis of Japanese Encephalitis Virus Infections with the Inbios JE *Detect*^™^ and DEN *Detect*^™^ MAC-ELISA Kits

**DOI:** 10.4269/ajtmh.15-0631

**Published:** 2016-04-06

**Authors:** Barbara W. Johnson, Christin H. Goodman, Youngmee Jee, David A. Featherstone

**Affiliations:** Diagnostic and Reference Laboratory, Arboviral Diseases Branch, Division of Vector-Borne Diseases, Centers for Disease Control and Prevention, Fort Collins, Colorado; Center for Immunology and Pathology, National Institute of Health, Korea Centers for Disease Control and Prevention, Seoul, Korea; Expanded Programme on Immunization, Regional Office for the Western Pacific, World Health Organization, Manila, Philippines

## Abstract

Japanese encephalitis virus (JEV) is the leading cause of pediatric viral neurological disease in Asia. The JEV-specific IgM antibody-capture enzyme-linked immunosorbent assay (MAC-ELISA) in cerebrospinal fluid (CSF) and serum is the recommended method of laboratory diagnosis, but specificity of JEV MAC-ELISA can be low due to cross-reactivity. To increase the specificity of the commercially available JE *Detect*^™^ MAC-ELISA (JE *Detect*), a differential testing algorithm was developed in which samples tested by JE *Detect* with positive results were subsequently tested by the DEN *Detect*^™^ MAC-ELISA (DEN *Detect*) kit, and results of both tests were used to make the final interpretation. The testing algorithm was evaluated with a reference panel of serum and CSF samples submitted for confirmatory testing. In serum, the false Japanese encephalitis (JE) positive rate was reduced, but sequential testing in CSF resulted in reduced JE specificity, as true JEV+ CSF samples had positive results by both JE *Detect* and DEN *Detect* and were classified as JE− (dengue virus [DENV]+). Differential diagnosis of JE by sequential testing with JE *Detect* and DEN *Detect* increased specificity for JE in serum, but more data with CSF is needed to make a final determination on the usefulness of this testing algorithm for CSF.

## Introduction

Japanese encephalitis virus (JEV) is the leading cause of viral neurological disease and disability in children under 15 years in Asia, with an estimated 68,000 cases annually, 20–30% of which are fatal.^1^ Approximately 30–50% of survivors have long-term sequelae.^2^ Neurological symptoms of JEV infection may be similar to those caused by other viral and bacterial pathogens, which makes laboratory-based diagnosis essential for guiding treatment or control strategies or both of this vaccine-preventable disease and other treatable infections.^3^

The JEV-specific IgM antibody-capture enzyme-linked immunosorbent assay (MAC-ELISA) is a sensitive method of laboratory diagnosis, as JEV IgM is produced soon after infection and is detectable in 90% of cases in cerebrospinal fluid (CSF) by 4 days and in serum by 7–9 days after the onset of clinical illness.^4^–^6^ However, JEV is a flavivirus and the specificity of MAC-ELISA for flaviviruses can be low due to IgM elicited to other flavivirus infections cross-reacting with the conserved immunogenic epitopes on the viral antigens used in the ELISA.^7^ Diagnosis by JEV MAC-ELISA alone can be problematic in some areas in Asia where JEV co-circulates with other flaviviruses such as dengue viruses (DENVs) and West Nile virus (WNV). In addition, DENV infections infrequently present with neurological symptoms similar to those of JEV, and dengue (DEN) cases have been included in acute encephalitis syndrome/acute meningoencephalitis syndrome (AES/AMES) surveillance studies.^8^,^9^

The JEV MAC-ELISA is recommended by the World Health Organization (WHO) to diagnose acute JEV infections and has been used by the WHO Japanese encephalitis (JE) laboratory network since 2006 for laboratory-based surveillance of JE and other causes of AES/AMES.^10^–^12^ Performance of three commercially available JEV MAC-ELISA kits has been assessed and recommendations to the JE laboratory network on their use has been guided by the results of these evaluations.^13^–^17^ The Panbio JE-Dengue IgM combo ELISA (Inverness Medical Innovations Inc., Queensland, Australia) was shown to have superior specificity compared with the Inbios JE *Detect*^™^ MAC-ELISA (JE *Detect*) (Inbios International Inc., Seattle, WA) and the JEV CheX (XCyton Diagnostics Ltd., Bangalore, India) kits because it used a JEV/DENV quantitative differential diagnostic testing algorithm. However, Panbio ceased manufacturing the kit in December 2013, and thus an alternative commercial assay or testing algorithm for differential diagnosis of JEV infections was needed.

Inbios International Inc. manufactures two separate MAC-ELISA kits for JE and DEN diagnosis, the JE *Detect* and DEN *Detect*^™^ MAC-ELISA (DEN *Detect*), respectively. Previously, the JE *Detect* kit was shown to have low specificity when DENV IgM+ samples were included in the evaluation sample set.^13^ However, it was observed at the Centers for Disease Control and Prevention (CDC) that the DEN *Detect* kit had high specificity when JEV IgM+ samples were included. We wanted to determine if the difference in specificity between the JE *Detect* and DEN *Detect* assays could be used to differentiate true JEV IgM positives from false positives (DENV IgM+). A JEV differential testing algorithm was developed in which samples tested by JE *Detect* with positive results were subsequently tested with the DEN *Detect* kit, and results of both tests used to make the final interpretation. Positive results in the less specific JE *Detect* test and negative results in the specific DEN *Detect* test would indicate the presence of JEV IgM only, whereas positive results in both tests would be interpreted as a false positive result by JE *Detect* cross-reacting with DENV IgM. The testing algorithm was evaluated with a reference panel comprised of JEV IgM+ and DENV IgM+ serum and CSF specimens, as well as a set of specimens collected during syndromic meningoencephalitis (ME) surveillance in Cambodia in 2013.

## Materials and Methods

### Specimens.

#### JE serological reference panel.

A panel of 200 sera (60 JEV IgM+, 24 DENV IgM+, five WNV IgM+, 111 JEV/DENV IgM−) and 75 CSF (24 JEV IgM+, nine DENV IgM+, and 42 JEV/DENV IgM−) was comprised of archived diagnostic specimens donated by JE reference and national network laboratories. The panel was first tested and samples classified at CDC by JEV and DENV MAC-ELISA and confirmed by JEV and DENV 90% plaque reduction neutralization assay (PRNT).^7^,^14^,^15^,^18^,^19^ A preliminary panel was sent to four reference laboratories for testing: the National Institute of Infectious Diseases (NIID), Japan; the National Institute of Mental Health and Neuro Sciences (NIMHANS), India; the U.S. Armed Forces Research Institute of Medical Sciences (AFRIMS), Thailand; and Universiti Malaysia Sarawak (UNIMAS), Malaysia. The inhouse assays of CDC, AFRIMS, and UNIMAS used a differential diagnostic testing algorithm that included both JEV and DENV MAC-ELISA.^19^–^21^ However, at AFRIMS and UNIMAS, CSF was tested only by the JEV IgM ELISA due to the limited sample volume; UNIMAS classified CSF as JE IgM+, non-JE flavivirus, and JE IgM−; and AFRIMS classified CSF as JE+ and JE−. Rather than an interpretation of equivocal (EQ), the NIMHANS assay classifies optical densities that are lower than the cutoff for JE but higher than the negative cutoff as non-JE flavivirus; and NIID used a JEV IgM assay only.^22^ Samples were scored as JEV IgM+ or JEV IgM−. DENV and WNV IgM+ samples were included in the JE IgM− subset. These samples may have had both JEV and DENV IgM+ results and were differentiated by PRNT (CDC) or comparison of quantitative units (AFRIMS and UNIMAS). Only samples in which at least four of the five reference laboratory results agreed were included in the final reference panel. No samples with EQ final results were included. Reference panels were prepared in 2011 with 45 μL volume and stored at −70°C until use.

#### Samples from Cambodia National Institute of Public Health.

National public health laboratories that participate in the WHO JE LabNet test samples using a validated in-house or commercial assay. All samples with JE positive and EQ results and about 10% of samples with negative results are sent to the WHO JE Global Specialized or Regional Reference laboratories for confirmatory testing as part of the quality assurance program. Sixty-four samples (25 CSF, 39 sera) without personal identifiers that had been collected from ME surveillance patients and tested in Cambodia from 2013 to 2014 with the Panbio kit were sent to CDC for confirmatory testing.^15^,^19^ Samples were tested by CDC JEV and DENV MAC-ELISA but not all were confirmed by PRNT due to the limited volume and necessary repeat testing. At CDC, seven serum samples had JEV+/DENV− results, four had JEV+/DENV EQ results, and one had a JEV+/DENV+ result. Because of the limited sample volume, PRNT was carried out with challenge viruses JEV, DENV-1, and DENV-2, as DENV-1 serotype was circulating in Cambodia at the time these samples were collected, and DENV-2 serotype may cross-react with the other DENV serotypes. This sample was confirmed as DENV by a 4-fold difference in neutralizing antibody titer against JEV compared with DENV in the PRNT. Twenty-five serum samples had negative results in both the CDC JEV and DENV MAC-ELISA. Two serum samples had JEV EQ results but no detectable neutralizing antibody titer, and were also classified as JEV IgM−.^15^ Of the 25 CSF samples tested by the CDC JEV and DENV MAC-ELISA the results were as follows: 15 JEV−/DENV−, three JEV+/DENV−, three JEV+/DENV EQ, and four JEV+/DENV+. One CSF sample with JEV+/DENV+ results was confirmed as JEV+ by PRNT; three had no detectable neutralizing antibody titer to JEV, DENV-1, or DENV-2 and could not be confirmed as either JEV or DENV. The three CSF with JEV+/DENV EQ results did not have sufficient volume remaining for PRNT. For these and other samples with JEV+/DENV+ results at CDC, agreement with CDC IgM ELISA results was the criteria, not final interpretation.

### Test methods.

CSF and serum sample panels were thawed once and thereafter stored at 4°C until the testing was completed. Sera were diluted 1:100 and tested according to the manufacturer's instructions in the JE *Detect* and DEN *Detect* kits. A note in the JE *Detect* kit insert states that CSF can be tested, but the kit had not been tested or optimized with CSF and before using the kit one has to optimize the CSF system. The DEN *Detect* instructions state that serum is the only sample type that should be used in the assay. Because of the limited sample volume, the CSF was diluted 1:10 and tested in single replicates in both assays. Samples with EQ results were retested, and those with EQ final results were coded as negative.

**Figure 1.**
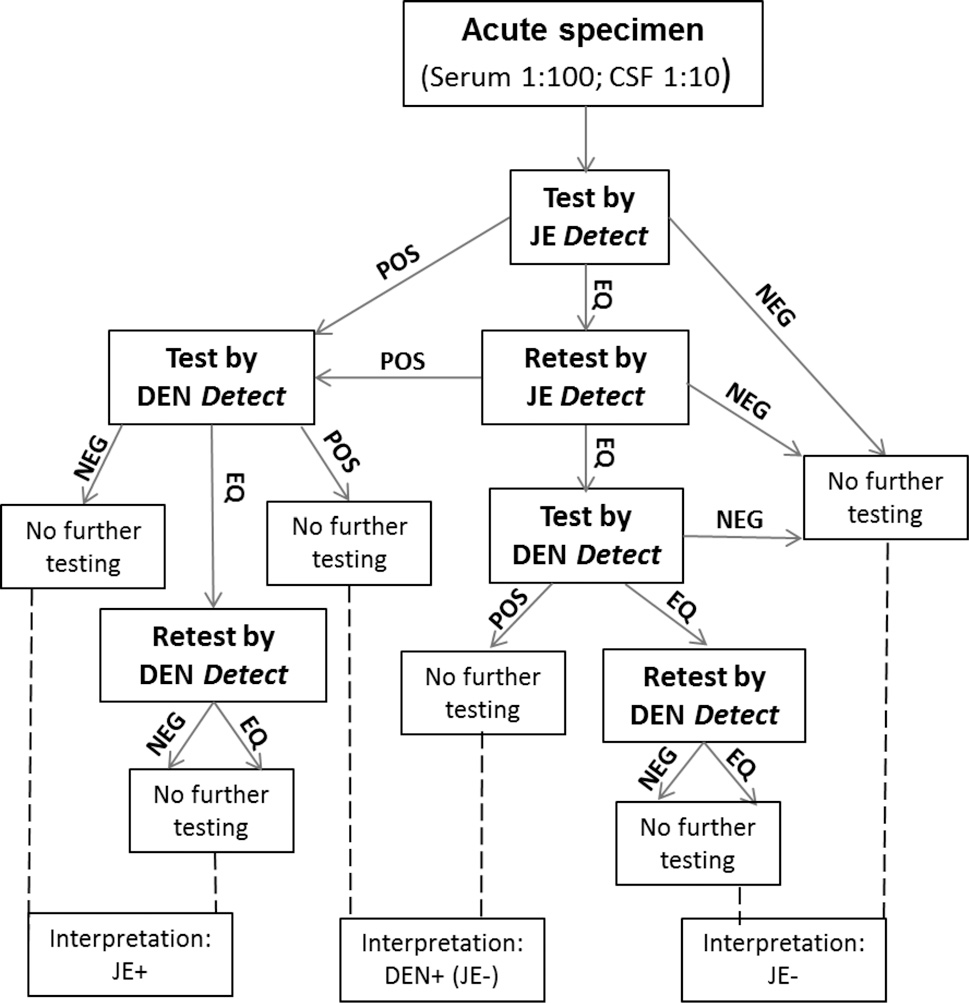
JE differential diagnostic testing algorithm using JE *Detect* and DEN *Detect* kits. Final interpretations, shown at the bottom of the figure, were made after all testing was completed. Samples with equivocal (EQ) results were retested and those that remained EQ after repeat testing were coded as NEG. DEN = dengue; DEN *Detect* = DEN *Detect*^™^ MAC-ELISA; DENV = dengue virus; EQ = equivocal; JE = Japanese encephalitis; JE *Detect* = JE *Detect*^™^ MAC-ELISA; JEV = Japanese encephalitis virus; MAC-ELISA = IgM antibody-capture enzyme-linked immunosorbent assay; EQ = equivocal; NEG = negative; POS = positive.

The testing algorithm and final interpretations are illustrated in Figure 1

. Briefly, all samples were tested first by the JE *Detect*. Samples with JEV+ results and those with JEV EQ results that remained EQ upon retesting were tested by DEN *Detect*. Samples with DENV EQ results were retested and EQ final results were coded as negative. Results were interpreted as shown in Table 1, with final classifications of JE+ or JE−. Samples with JEV+ and DENV− or EQ results were interpreted as JE; samples with JEV+ or EQ results and DENV+ results were interpreted as DEN and classified as JE−; samples with JEV EQ and DENV− or EQ results were interpreted as negative and classified as JE−.

## Results

### Reference serum testing.

**Figure 2.**
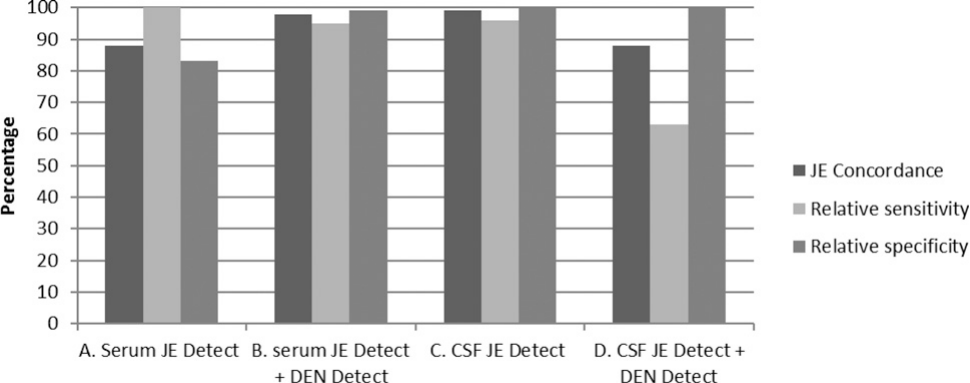
Summary of the comparison of test results of a JE reference serological panel tested either by JE *Detect* kit alone or sequentially by JE *Detect* and DEN *Detect* according to the testing algorithm in Figure 1. CSF = cerebrospinal fluid; DEN *Detect* = DEN *Detect*^™^ MAC-ELISA; JE = Japanese encephalitis; JE *Detect* = JE *Detect*^™^ MAC-ELISA; MAC-ELISA = IgM antibody-capture enzyme-linked immunosorbent assay.

The 200 serum specimens were classified by reference laboratory testing as JE+ (*N* = 60) or JE− (*N* = 140); reference DENV IgM+ (*N* = 24) and WNV IgM+ (*N* = 5) samples were included in the JE− subset. All samples were tested by JE *Detect*, and those with EQ results were retested (Table 2). Of the 60 reference JEV IgM+ serum samples, 60 also had positive results in the JE *Detect* assay. Of 24 reference DENV IgM+ sera, 23 had positive results by JE *Detect* including two samples with JEV EQ results, which were JEV+ on retesting. One reference DENV IgM+ sample with JEV EQ results remained JEV EQ after retesting. Four of the five reference WNV IgM+ samples (JE−) were negative and one of five was positive by JE *Detect*. Of the 111 reference JEV/DENV/WNV IgM− samples, 109 had negative results by JE *Detect*, including four that were initially JEV EQ; two samples had JEV EQ results in replicate testing. In summary, based on testing only by JE *Detect*, with JEV EQ results coded as negative, concordance with reference results was 88%, and relative sensitivity and specificity was 100% and 83%, respectively (Figure 2A

).

The 87 sera with JEV+ (*N* = 84) or EQ (*N* = 3) results in JE *Detect* were later tested DEN *Detect* (Table 2). The 113 sera with negative results by JE *Detect* were not tested further and correctly classified as JE−. Of the 60 reference JEV IgM+ samples, which were also JEV+ in the JE *Detect* assay, 57 had DENV− final results and were correctly classified as JE+, including 14 samples with DENV EQ results after retesting coded as DENV−. Three JE+ serum samples with positive results in the reference and JE *Detect* assays had DENV+ results in the DEN *Detect* assay, and were therefore interpreted as DEN and classified as JE−. All 24 of the reference DENV IgM+ sera, which had JEV+ (*N* = 23) or EQ (*N* = 1) results by JE *Detect*, were also positive by DEN *Detect* and were interpreted as DEN and correctly classified as JE−. Two reference JEV IgM− samples with JEV EQ results by JE *Detect* had negative results by DEN *Detect* and were coded as NEG (negative), and correctly classified as JE−. The reference WNV IgM+ sample with a JEV+ result by JE *Detect* had a negative result by DEN *Detect*, and was therefore incorrectly interpreted as JE. Final interpretations of the 200 serum samples tested sequentially by JE *Detect* and DEN *Detect* kits, based on the testing algorithm in Figure 1 and classified as JE+ or JE− according to Table 1, are summarized in Table 2 and Figure 2B. Concordance between reference test results and those from sequential testing by JE *Detect* and DEN *Detect* kits increased to 98%; relative sensitivity for JE decreased slightly to 95%. Relative specificity for JE increased to 99%, as the 24 reference DENV+ sera with JEV+ results in JE *Detect* also had positive results by DEN *Detect*, and according to the interpretations in Table 1, they were correctly classified as JE−. Had these samples only been tested by JE *Detect* they would have been incorrectly classified as JE+.

### Reference CSF testing.

The 75 CSF samples were classified by reference laboratories as JE+ (*N* = 24) and JE− (*N* = 51), including nine CSF that had been confirmed as DENV IgM+ by PRNT at CDC, but which were not tested by the other reference laboratories for DENV IgM. Results of testing by JE *Detect* are shown in Table 2. Of the 24 reference JEV IgM+ CSF samples, 23 were JEV+ and one was EQ after retesting by JE *Detect*. Seven of nine CDC DENV+ CSF were JEV− in JE *Detect* and two of nine CSF had JEV EQ initial results and EQ and JEV− results after retesting. All 42 of the reference JEV/DENV IgM− CSF also had negative results in JE *Detect* and were not tested further. Based on testing only by JE *Detect*, and coding EQ results as negative, concordance was 99%, sensitivity was 96%, and specificity was 100% (Figure 2C).

The 25 CSF with JEV IgM+ (*N* = 23) or EQ (*N* = 2) results by JE *Detect* were subsequently tested by DEN *Detect* (Table 2). Fifteen of 24 reference JEV IgM+ samples had DENV− or EQ results, and were interpreted as JE and eight of 24 had positive results by both JE *Detect* and DEN *Detect* and were interpreted as DEN and incorrectly classified as JE−. The CSF with a JEV EQ result by JE *Detect* had a negative result in the DEN *Detect* assay and was incorrectly classified as JE−. The CDC DENV IgM+ CSF sample with EQ result by JE *Detect* had a NEG result by DEN *Detect* and was classified correctly as JE−. According to the testing algorithm, the other eight CDC DENV IgM+ CSF with JEV− results by JE *Detect* would not be tested by DEN *Detect*. However, to determine the sensitivity of DEN *Detect* for CSF samples, all the CDC DENV IgM+ CSF were tested by DEN *Detect* and all had negative results. Final interpretations of the 75 CSF samples tested sequentially by JE *Detect* and DEN *Detect* kits, based on the testing algorithm in Figure 1 and classified as JE+ or JE− according to Table 1, are summarized in Table 2. JE concordance decreased to 88%, JE sensitivity decreased to 63%, and JE specificity remained at 100% when the DEN *Detect* results were factored into the interpretations (Figure 2D).

### Testing samples from Cambodia National Institute of Public Health.

**Figure 3.**
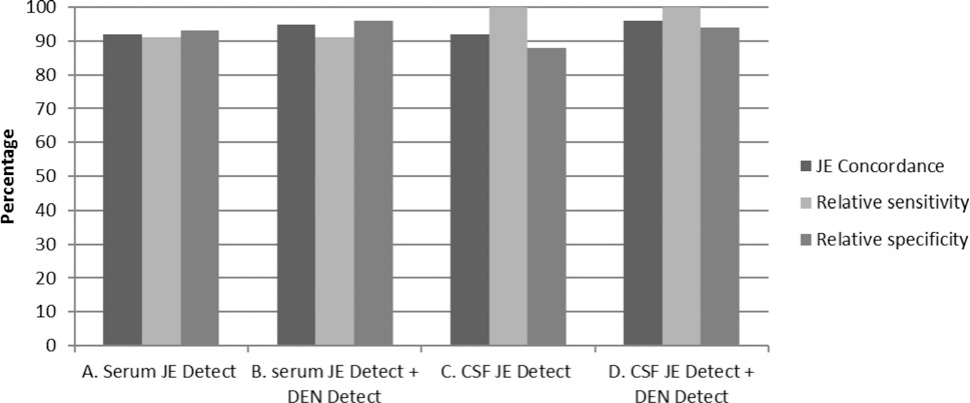
Summary of the comparison of test results for 64 samples from Cambodia to CDC JEV and DENV MAC-ELISA results as the reference standard. Samples were tested either by JE *Detect* kit alone or were sequentially tested by JE *Detect* and DEN *Detect* according to the testing algorithm in Figure 1. CDC = Centers for Disease Control and Prevention; CSF = cerebrospinal fluid; DEN *Detect* = DEN *Detect*^™^ MAC-ELISA; DENV = dengue virus; JE = Japanese encephalitis; JE *Detect* = JE *Detect*^™^ MAC-ELISA; MAC-ELISA = IgM antibody-capture enzyme-linked immunosorbent assay.

A total of 64 samples (39 sera, 25 CSF) were submitted to CDC for confirmatory testing from Cambodia as part of the JE laboratory network quality assurance program. These samples had been tested with the discontinued Panbio kit in Cambodia and therefore these results were not considered. At CDC, samples were tested simultaneously by CDC JEV and DENV MAC-ELISA then sequentially by JE *Detect* and DEN *Detect* according to the testing algorithm (Figure 1). Because of the limited sample volume, PRNT with JEV, DENV-1, and DENV-2 was only carried out on seven samples with discordant results after the ELISA testing was completed. Ten of 11 of the CDC JEV IgM+ serum samples were correctly classified as JE+ by JE *Detect* testing alone (Table 3) and by sequential testing with JE *Detect* and DEN *Detect* (Table 3). One serum sample with JEV/DENV IgM+ results at CDC, confirmed as DENV+ by PRNT, also had positive results in the JE *Detect* and DEN *Detect* tests and was interpreted as DEN and correctly classified JE−. One of 27 serum samples with negative results at CDC had JEV+ and DENV− results by JE *Detect* and DEN *Detect* tests and was incorrectly classified as JE+. Concordance between CDC reference results and those from testing only by JE *Detect*or or sequentially by JE *Detect* and DEN *Detect* was 92% and 95%, respectively; relative sensitivity for both methods was 91%; and specificity was 93% and 96% (Figure 3

).

Results of testing 25 CSF by CDC JEV and DENV MAC-ELISA were as follows: 15 JEV−/DENV−, three JEV+/DENV−, three JEV+/DENV EQ, and four JEV+/DENV+. PRNT was done on the four JEV+/DENV+ CSF; one of four had neutralizing titer to JEV, but not to DENV 1 and 2 and was classified as JE+. This sample had JEV+ and DENV EQ results in JE *Detect* and DEN *Detect* tests and was correctly interpreted as JE. The other three CSF had no detectable neutralizing antibody to either JEV or DENV. Therefore, for these three CSF samples, either JEV+ or DENV+ results in the JE *Detect* and DEN *Detect* were considered correct and concordant with CDC results. Under these criteria, one of three CSF samples had JEV+ and DENV+ results by JE *Detect* and DEN *Detect* testing, respectively, and was interpreted as DEN (JE−) and scored as concordant. Two of three CSF had JEV+/DENV EQ results by JE *Detect* and DEN *Detect* and were interpreted as JE and were concordant with CDC results (Table 3). The three CDC JEV+/DENV− and three CDC JEV+/DENV EQ CSF were also JEV+ and DENV− in the JE *Detect* and DEN *Detect* tests, respectively, and were interpreted as JE and concordant with CDC results. In summary, a total of nine CSF were classified as JE+ and one was interpreted as DEN (JE−) (Table 3). One of the 15 CSF with CDC JEV−/DENV− results was JEV+ and DENV EQ by JE *Detect* and DEN *Detect* tests, respectively, and was incorrectly interpreted as JE. Concordance between CDC results and those from testing only by JE *Detect* or sequentially by JE *Detect* and DEN *Detect* was 92% and 96%, respectively. Relative sensitivity was 100% for both testing strategies and relative specificity was 88% and 94% (Figure 3).

## Discussion

JE is a vaccine-preventable disease, in contrast to DEN and WN, for which there are no effective control or treatment strategies. Accurate laboratory diagnosis of JEV infections and differentiation from other flaviviruses with similar clinical presentation is essential for public health decision making. JEV-specific MAC-ELISA is more sensitive than JEV RNA detection, due to the low-level, transient viremia that is generally cleared by the onset of illness, and the rapid elicitation of JEV-specific IgM early in acutely ill patients.^4^–^6^ CSF is the preferred specimen for JE diagnosis, as IgM in serum elicited against other flavivirus infections does not cross into the CSF.^4^,^6^ Also, in cases of neuroinvasive disease, antibodies in serum may not be the cause of the neurological disease. However, a single acute serum sample is often the only specimen type obtained as it is often not feasible or safe to collect CSF in clinical settings.

Because of the cross-reactivity of IgM antibodies to the conserved immunogenic epitopes on the flavivirus envelope protein, specificity of JEV MAC-ELISA can be low, particularly in areas where multiple flaviviruses co-circulate and the population is likely exposed.^7^,^9^,^13^,^15^ This makes laboratory diagnosis of JEV infection with a single specimen challenging, even in a reference laboratory. At CDC, the PRNT is used to confirm MAC-ELISA results and also as a more specific test to differentiate flavivirus cross-reactivity in primary flavivirus infections.^19^ However, in areas where flaviviruses co-circulate and most flavivirus infections are secondary infections, neutralizing antibodies from past infections also cross-react in the PRNT and confound differential diagnostic test interpretation. In addition, the PRNT is expensive and technically difficult and thus not a practical test for most laboratories with limited resources.

One strategy for reducing flavivirus cross-reactivity is to make a recombinant antigen in which the consensus epitopes are removed. However, these cross-reactive epitopes are also highly immunogenic and removing them may reduce antigen reactivity, and subsequently, sensitivity. Another method of differential diagnostic testing is a quantitative ELISA, in which the IgM titer is calculated into units and each flavivirus in the test is calibrated against the others, such as in the Panbio JE/DEN Combo ELISA and AFRIMS in-house assay.^4^,^20^ When Panbio announced that it would no longer manufacture the kit, and in the absence of an alternate commercial JEV IgM detection assay with similar specificity, another strategy of JE differential diagnosis was needed, particularly in the JE LabNet countries where JEV and DENV co-circulate and DEN cases are included in AMES surveillance.

Inbios developed and optimized two separate MAC-ELISA assays, JE *Detect* and DEN *Detect*. Although there are some common components, the dilution buffers are different and cannot be substituted for the other (data not shown), they have been calibrated separately, and they have different cutoff values. However, the formats and calculations of the tests are the same and both use a recombinant virus-like particle containing the structural membrane and envelope proteins.^23^ Neither kit has been optimized for CSF, although CSF has been tested and evaluated in the JE *Detect*.^15^ We wanted to determine if by testing samples sequentially, the specificity of the JE *Detect* could be improved. A testing algorithm was developed in which all samples were first tested by JE *Detect* but only samples with positive or EQ results were tested with the DEN *Detect* kit. This was more economical than testing all specimens by both tests simultaneously. In addition, the specificity criterion was JE+ or JE−; samples with JEV− results that were true DENV or other flavivirus infections were not considered.

The majority of serum specimens in the well-characterized reference panel were correctly identified by testing initially with the non–JE-specific JE *Detect*, then with the more specific DEN *Detect*. Had the DEN *Detect* testing not been done, 23 of 24 of reference DENV IgM+ sera which were JEV+ in JE *Detect*, would have been have been interpreted as JE.

There is considerable cross-reactivity in the CDC JEV and DENV MAC-ELISA, which is why the PRNT is an essential confirmatory test in the CDC differential diagnostic testing algorithm (Table 4). Thirteen of the 24 reference JEV IgM+ CSF had JEV/DENV IgM+ results at CDC and were confirmed as JE+ only by PRNT (4-fold higher neutralization titer for JE). Included in this subset were the eight CSF with JEV+/DEN+ results by JE *Detect* and DEN *Detect* testing, respectively (Table 4). Based on CDC JEV and DENV MAC-ELISA results only, the Inbios JE *Detect* and DEN *Detect* assays had higher specificity for JE. Twelve of 12 JE+ samples with CDC JEV+/DENV− results and three of 12 JE+ samples with CDC JEV+/DEN+ results (interpreted as indeterminate) were correctly classified as JE+ by JE *Detect* and DEN *Detect* testing (Table 5). Eight of the nine CDC DEN+ samples were correctly classified as JE− by JE *Detect* and DEN *Detect* testing, including three samples with CDC JEV+/DENV+ results. However, use of the testing algorithm for CSF in some cases confounded diagnosis. DEN *Detect* was not specific or sensitive for DENV+ CSF, as eight of the 24 reference JEV IgM+ CSF had positive results in DEN *Detect* and were interpreted as DEN and classified as (false) JE− and none of the nine CSF confirmed as DENV+ by PRNT at CDC were detected by DEN *Detect* (Table 4). In contrast, JE *Detect* had high sensitivity, with 23 of 24 reference JEV IgM+ CSF having a positive result. In addition, there was less cross-reactivity in the JE *Detect* assay to DENV+ CSF compared with serum samples: one of nine CSF compared with 23 of 24 in serum. With only eight CDC-confirmed DENV+ CSF samples, conclusions could not be made about the accuracy of the testing algorithm with CSF samples.

The reference panel was comprised of specimens that had been well-characterized and confirmed at CDC by PRNT. Samples in which a final determination could not be made, or those with results that were discordant between the reference laboratories were excluded. Evaluation of the testing algorithm was needed with the kind of samples that are tested in the JE laboratory network; i.e., those collected in surveillance programs or based on clinical diagnosis. The samples from Cambodia illustrate the difficulty of flavivirus diagnosis even in reference laboratories. Many of the specimens had JEV+/DENV+ results in the CDC MAC-ELISA, but few could be confirmed by PRNT. This was probably because JE patients, most of them children, are acutely ill and medical care is sought within the first day or so of onset of illness, before the neutralizing titer rises to detectable levels. Only one serum and one CSF sample were confirmed by PRNT as DEN+ (JE−). These samples did have positive results in the JE *Detect* test, but were correctly identified as DEN by DEN *Detect*, thus concordance and specificity with CDC results increased as well.

Although DEN diagnosis generally is not the focus of AMES surveillance and DENV infections typically have different clinical presentations from neuroinvasive JEV infections, it has been shown that DEN cases are included in AMES surveillance either due to the broad clinical definition of AMES, or because neuroinvasive DENV infections occur more frequently than previously thought.^8^ Implementation of the differential testing algorithm with the JE *Detect* and DEN *Detect* kits may reduce the number of false JE-positive results reported due to cross-reactivity with DEN cases, particularly in serum. However, because the numbers of DEN IgM+ CSF samples in these evaluations was small and the DEN *Detect* kit is not intended for CSF testing, further investigation is warranted before any conclusions can be made on the usefulness of the DEN *Detect* kit for differential diagnosis of JEV infections with CSF samples.

## Figures and Tables

**Table 1:** Interpretation of differential diagnostic testing results with sequential testing by JE *Detect* and DEN *Detect* kits

JE result	DEN result	Final interpretation	Classification
JEV+	DENV−	JE	JE+
JEV+	DENV+	DEN	JE−
JEV+	DENV EQ (after retest)	JE	JE+
JEV EQ (after retest)	DENV−	NEG	JE−
JEV EQ (after retest)	DENV+	DEN	JE−
JEV EQ (after retest)	DENV EQ (after retest)	NEG	JE−
JEV−	Not Tested	NEG	JE−

DEN *Detect* = DEN *Detect*^™^ MAC-ELISA; DENV = dengue virus; EQ = equivocal; JE = Japanese encephalitis; JE *Detect* = JE *Detect*^™^ MAC-ELISA; JEV = Japanese encephalitis virus; MAC-ELISA = IgM antibody-capture enzyme-linked immunosorbent assay; NEG = negative.

**Table 2:** Test results for JEV reference panel samples tested by JE *Detect* or sequentially by JE *Detect* and DEN *Detect* kits compared with reference laboratory results

Test	Interpretation/classification*	Reference JEV IgM+ (JE+)	Reference DENV IgM+ (JE−)	Reference JEV/DENV IgM− (JE−)†	Total
Serum					
JE *Detect*	JE/JE+	60	23	1‡	84
	NEG/JE−	0	0	113	113
	JEV EQ	0	1	2	3
	Total	60	24	116	200
Serum					
JE *Detect* + DEN *Detect*	JE/JE+	57	0	1‡	58
	DEN/JE−	3	24	0	27
	NEG/JE−	0	0	2	2
	Total	60	24	3	87
CSF					
JE *Detect*	JE/JE+	23	0	0	23
	NEG/JE−	0	8	42	50
	JEV EQ	1	1	0	2
	Total	24	9	42	75
CSF					
JE *Detect* + DEN *Detect*	JE/JE+	15	0	0	15
	DEN/JE−	8	0	0	8
	NEG/JE−	1	9	0	10
	Total	24	9	0	33

CSF = cerebrospinal fluid; DEN = dengue; DEN *Detect* = DEN *Detect*^™^ MAC-ELISA; DENV = dengue virus; JE *Detect* = JE *Detect*^™^ MAC-ELISA; EQ = equivocal; JE = Japanese encephalitis; JEV = Japanese encephalitis virus; MAC-ELISA = IgM antibody-capture enzyme-linked immunosorbent assay; NEG = negative.

* Based on Table 1; EQ results after retesting coded as NEG.

† Including five reference WNV IgM+ samples.

‡ Reference WNV IgM+.

**Table 3:** Comparison of test results for 64 samples from Cambodia NIPH tested sequentially by JE *Detect* and DEN *Detect* kits to CDC JEV and DENV MAC-ELISA results

	Interpretation/classification*	CDC JEV IgM+	CDC JEV/DENV IgM+†	CDCJEV/DENV IgM−	Total
Serum					
JE *Detect*	JE/JE+	10	1	1	12
	NEG/JE−	0	0	26	26
	JEV EQ	1	0	0	1
	Total	11	1	27	39
Serum					
JE *Detect* + DEN *Detect*	JE/JE+	10	0	1	11
	DEN/JE−	0	1	0	1
	NEG/JE−	1	0	0	26
	Total	11	1	1	
CSF					
JE *Detect*	JE/JE+	9	1	1	11
	NEG/JE−	0	0	14	14
	JEV EQ	0	0	0	0
	Total	9	1	15	25
CSF					
JE *Detect* + DEN *Detect*	JE/JE+	9	0	1	10
	DEN/JE−	0	1	0	1
	NEG/JE−	0	0	0	0
	Total	9	1	1	11

CDC = Centers for Disease Control and Prevention; CSF = cerebrospinal fluid; DEN *Detect* = DEN *Detect*^™^ MAC-ELISA; DENV = dengue virus; EQ = equivocal; JE = Japanese encephalitis; JE *Detect* = JE *Detect*^™^ MAC-ELISA; JEV = Japanese encephalitis virus; MAC-ELISA = IgM antibody-capture enzyme-linked immunosorbent assay; NEG = negative; PRNT = plaque reduction neutralization assay.

* Interpretations based on Table 1; EQ results after re-testing coded as NEG.

† Confirmed as DENV+ by plaque reduction neutralization test.

**Table 4:** Complete test results for 33 reference CSF classified as JE+ or DEN (JE−) at CDC

Sample number	CDC JEV P/N	CDC DENV P/N	JEV PRNT	DENV PRNT	CDC final interpretation*	JE *Detect* ISR	JE *Detect* results†	DEN *Detect* ISR	DEN *Detect* results‡	Inbios final interpretation
1	18.93	1.23	1:640	1:160	JE+	64.22	JE	2.20	EQ	JE+
2	20.08	1.57	1:4	< 1:4	JE+	44.83	JE	1.50	NEG	JE+
3	17.66	1.67	1:4	< 1:4	JE+	46.83	JE	1.15	NEG	JE+
4	11.93	1.80	1:8	< 1:8	JE+	67.15	JE	1.23	NEG	JE+
5	19.4	3.83	1:640	1:160	JE+	75.84	JE	3.3	DEN+	DEN+
6	19.07	3.30	1:8	< 1:4	JE+	83.81	JE	2.25	EQ	JE+
7	20.80	3.53	1:4	< 1:4	JE+	4.68 (4.1)	EQ	1.108	NEG	NEG
8	19.60	10.76	1:8	< 1:8	JE+	52.70	JE	6.14	DEN+	DEN+
9	31.02	5.41	1:8	< 1:8	JE+	86.17	JE	3.20	DEN+	DEN+
10	22.25	4.64	1:160	1:40	JE+	79.30	JE	2.54	EQ	JE+
11	24.11	9.17	1:320	1:80	JE+	74.34	JE	3.37	DEN+	DEN+
12	22.64	7.64	1:16	< 1:4	JE+	79.33	JE	3.6	DEN+	DEN+
13	18.87	6.91	1:16	< 1:4	JE+	68.67	JE	2.3 (2.14)	EQ	JE+
14	20.68	1.95	1:8	< 1:4	JE+	73.40	JE	1.36	NEG	JE+
15	21.68	3.10	1:4	< 1:4	JE+	79.58	JE	1.53	NEG	JE+
16	27.3	6.16	1:16	< 1:4	JE+	85.45	JE	3.76	DEN+	DEN+
17	10.87	14.52	1:320	< 1:20	JE+	84.29	JE	3.34	DEN+	DEN+
18	29.42	7.40	1:160	< 1:20	JE+	87.49	JE	2.8 (4.15)	EQ/DEN+	DEN+
19	6.72	1.20	1:320	< 1:20	JE+	82.74	JE+	1.35	NEG	JE+
20	6.41	1.77	1:320	1:20	JE+	48.05	JE	0.96	NEG	JE+
21	4.91	1.98	1:320	< 1:20	JE+	39.63	JE	1.26	NEG	JE+
22	3.56	1.68	1:80	< 1:20	JE+	6.86	JE	2.22 (2.51)	EQ	JE+
23	3.89	1.00	1:4	< 1:4	JE+	27.37	JE	1.07	NEG	JE+
24	3.99	0.94	1:8	< 1:8	JE+	53.11	JE	1.08	NEG	JE+
25	1.58	2.02	1:40	1:320	DEN+	4.57 (4.63)	EQ	1.03	NEG	NEG
26	1.44	2.01	< 1:2	1:320	DEN+	2.40	NEG	1.02	NEG	NEG
27	1.24	3.52	< 1:8	1:8	DEN+	2.97	NEG	1.44	NEG	NEG
28	1.799	3.92	< 1:4	1:4	DEN+	1.93	NEG	1.02	NEG	NEG
29	4.08	5.57	< 1:4	> 1:128	DEN+	3.84	NEG	1.52	NEG	NEG
30	2.84	2.15	< 1:4	1:32	DEN+	3.71	NEG	1.03	NEG	NEG
31	1.02	3.00	< 1:8	1:8	DEN+	4.8 (2.76)	EQ/NEG	2.79 (2.24)	EQ	NEG
32	2.32	3.46	< 1:4	1:4	DEN+	2.38	NEG	1.14	NEG	NEG
33	1.50	2.96	< 1:4	1:4	DEN+	2.05	NEG	1.16	NEG	NEG

CDC = Centers for Disease Control and Prevention; CSF = cerebrospinal fluid; DEN = dengue; DEN *Detect* = DEN *Detect*^™^ MAC-ELISA; DENV = dengue virus; EQ = equivocal; ISR = Inbios Detect immune status ratio; JE = Japanese encephalitis; JE *Detect* = JE *Detect*^™^ MAC-ELISA; JEV = Japanese encephalitis virus; MAC-ELISA = IgM antibody-capture enzyme-linked immunosorbent assay; NEG = negative; P/N = mean OD of sample reacted on viral antigen/mean OD of negative control sample reacted on viral antigen.

* CDC ELISA interpretations: NEG P/N < 2; EQ P/N 2–3; POS, P/N > 3.

† Calculation of JE Detect results: NEG, ISR < 4.0; EQ, ISR 4–6; JE+, ISR > 6.0.

‡ Calculation of DEN Detect results: NEG, ISR < 1.65; EQ, ISR 1.65–2.84; DEN+, ISR > 2.84.

**Table 5:** Comparison of CDC and Inbios CSF test results based on JEV and DENV MAC-ELISA testing only

		CDC JE+		CDC DEN+		CDC NEG	Total
		JEV+/DENV−	JEV+/DEN+*	JEV−/DENV+	JEV+/DEN+*	JEV/DEN−	
JE *Detect* + DEN *Detect*	Inbios JE+/DEN−	12	3†	1	0	0	16
	Inbios JE+/DEN+	0	8	0	0	0	8
	Inbios JE−/DEN−	0	1	5	3	42	51
	Total	12	12	6	3	42	75

CDC = Centers for Disease Control and Prevention; CSF = cerebrospinal fluid; DEN *Detect* = DEN *Detect*^™^ MAC-ELISA; DENV = dengue virus; EQ = equivocal; JE = Japanese encephalitis; JE *Detect* = JE *Detect*^™^ MAC-ELISA; JEV = Japanese encephalitis virus; MAC-ELISA = IgM antibody-capture enzyme-linked immunosorbent assay; NEG = negative.

* Differential diagnosis determined by 4-fold higher neutralization titer.

† DEN *Detect* EQ results were coded as NEG.
